# Co-infection by *Streptococcus anginosus *and *Mycobacterium tuberculosis*: three case reports

**DOI:** 10.1186/1752-1947-3-37

**Published:** 2009-01-29

**Authors:** Ramón Rabuñal, Juan Corredoira, Rafael Monte, Amparo Coira

**Affiliations:** 1 Department of Internal Medicine, Complexo Hospitalario Xeral-Calde, Severo Ochoa, Lugo, Spain; 2 Department of Microbiology, Complexo Hospitalario Xeral-Calde, Severo Ochoa, Lugo, Spain

## Abstract

**Introduction:**

Bacterial infections may appear as sequelae of remote tuberculous infections, especially thoracic infections. The simultaneous appearance of tuberculosis and bacterial infection is not common, and, to our knowledge, the association of infection by *Streptococcus anginosus *and *Mycobacterium tuberculosis *has not been reported previously in the literature.

**Case presentation:**

We report three cases of dual infection with *Streptococcus anginosus *and *Mycobacterium tuberculosis *that were first diagnosed as pyogenic abscesses because of an isolation of *Streptococcus anginosus*. Despite a course of antibiotics and drainage, the outcome of this initial treatment was unfavourable. A re-evaluation yielded a diagnosis of mixed infection with *Streptococcus anginosus *and *Mycobacterium tuberculosis*.

**Conclusion:**

In a geographical area with a high prevalence of tuberculous disease, the rare possibility of dual infection with *Streptococcus anginosus *and *Mycobacterium tuberculosis *should be considered.

## Introduction

The simultaneous appearance of tuberculosis and bacterial infection is not common. It has been described mainly in patients with acquired immunodeficiency syndrome, presenting with co-infection with tuberculosis and pneumococcal pneumonia [1]. The *Streptococcus anginosus *group (SAG) includes three well-differentiated species: *Streptococcus constellatus, S. anginosus *and *S. intermedius*. The simultaneous and clinically significant isolation of SAG and *Mycobacterium tuberculosis *in a single site of infection has not previously been described to our knowledge. Cases that have been described are in patients with thoracic infection due to SAG over residual tuberculous thoracic lesions, as well as an association of simultaneous SAG pericarditis and lung tuberculosis [2,3]. Here, we describe three cases of abscesses with dual infection by SAG and *Mycobacterium tuberculosis.*

## Case presentation

### Case 1

A 40-year-old man, diagnosed with Addison's disease 2 years before, presented with a 1-month history of intermittent fever and no other relevant symptoms. His physical examination revealed no abnormalities. A haemogram, a routine biochemistry profile and coagulant tests were all normal, except for an erythrocyte sedimentation rate of 70 mm/hour. Radiographs of the chest and abdomen and an echocardiogram were unhelpful. *Brucella spp *serology and blood cultures were negative. Mantoux (5 UI) was 12 mm. Ziehl-Neelsen stain and mycobacterial urine cultures were negative, as was a bone-marrow culture for *Mycobacteria *and *Brucella*. An abdominal computer tomography (CT) scan showed a hypodense mass in the right lobe of the liver and an enlarged right adrenal gland with calcifications. Fine-needle aspiration of the liver was negative for malignant cells. Microscopic examination of stained specimens revealed no acid-fast bacilli or other microorganisms, and cultures (aerobic and anaerobic) for bacteria and fungi were negative. Laparotomy was performed, obtaining purulent material from the adrenal gland, with extension to the liver and duodenum. Gram and Ziehl-Neelsen stains of the pus were negative, but *Streptococcus constellatus *was isolated in pure culture, using the API 20S system. The minimum inhibitory concentration (MIC) for penicillin was 0.03 microgr/ml. Microscopic examination revealed granulomas in the liver and adrenal tissue. The patient was treated with ceftriaxone, rifampicin, isoniazid and pyrazinamide, and his condition improved. Löwenstein culture of the abscess material yielded positive result for *Mycobacterium tuberculosis*.

### Case 2

A 68-year-old woman was admitted to hospital with general malaise, arthromyalgia and fever lasting for 2 months. Ten days prior to admission, she started experiencing dorsolumbar pain radiating to the right rib region. The only abnormalities observed on physical examination were temperature of 37.5°C and pain on palpation of the spinal thoracic apophyses 11 and 12. The results of her blood analysis were as follows: haemoglobin 13.4 gr/dl, 7100 leucocytes/mm3, erythrocyte sedimentation rate 39 mm/hour and albumin 3.3 gr/dl. Other blood tests results were normal. Mantoux was 25 mm (5 UI). *Brucella *serology and blood cultures were both negative. An echocardiogram showed hypertensive cardiomyopathy. Radiography of the spinal column revealed destruction and crushing of thoracic vertebrae 10 and 12, with an adjacent paravertebral mass. Spinal CT scan showed destructive and erosive lesions in those vertebrae, involving the intervetebral discs and an adjacent paravertebral mass.

CT-guided fine-needle aspiration of the paravertebral mass was performed. Purulent material was extracted, for which both Gram and Ziehl-Neelsen stains were negative. Subsequently, *Streptococcus constellatus *was isolated in pure culture, using the API 20S system. MIC for penicillin was 0,06 microgr/ml. The patient was treated with penicillin with initial clinical improvement. After 2 months of treatment, her condition suddenly worsened both clinically and radiologically. On physical examination, two soft lumps were detected in the left paravertebral region near the level of thoracic vertebra 11 and pus was extracted. Gram stain revealed no microorganisms and Ziehl-Neelsen stain showed acid-fast bacilli. Concurrently, the culture from the needle aspiration performed during the first admission was received, with an isolation of *Mycobacterium tuberculosis*. Treatment was initiated with rifampicin, isoniazid and pyrazinamide, resulting in clinical improvement. One month later, the patient was admitted to the hospital with acute myocardial infarction and died a few hours later.

### Case 3

A 70-year-old woman presented with a constitutional syndrome lasting 2 months with pain in the lumbar region and a limp. Physical examination revealed temperature of 38°C and pain on flexing the left hip but no other abnormal findings. Blood analysis showed: haemoglobin 11 gr/dl, 13800 leucocytes/mm^3 ^(75% neutrophils), 380000 platelets/mm^3^; erythrocyte sedimentation rate 109 mm/hour, fibrinogen 981 mg/dl, albumin 2.9 gr/dl and ferritin 559 ng/ml. Radiography of the abdomen showed blurring of the line of the left psoas. Abdominal CT scan revealed a mass of lower density in the left psoas.

Percutaneous drainage was placed in the above-mentioned area, and purulent material was extracted. Gram-positive cocci in chains were observed, while a Ziehl-Neelsen stain was negative. Subsequently, *Streptococcus anginosus *was isolated in pure culture, using the API 20S system. MIC to penicillin was 0.03 microgr/ml. Treatment with ceftriaxone was initiated and the drainage maintained for 1 week.

When a new CT scan conducted after two weeks of treatment showed persistence of the abscesses, surgical drainage was indicated. Forty-five days after admission, Löwenstein culture from the previous puncture yielded a positive result for *Mycobacterium tuberculosis*. Treatment with isoniazid, rifampicin and pyrazinamide was initiated, after which the patient's condition improved both clinically and radiologically.

## Discussion

Tuberculous abscesses are infrequent in immunocompetent patients, especially since the advent of tuberculostatic drugs, and they are usually secondary to spinal involvement [4]. Dual infection of an abscess with bacteria and *Mycobacterium tuberculosis *is known but rarely described [5]. The SAG are common inhabitants of the digestive tract, characterized by a special tendency to produce purulent diseases and abscess formation [6]. In suppurative infections, SAG is isolated in association with other microorganisms from gastrointestinal flora in almost half of all cases. The pathogenicity of SAG strains may be enhanced by the co-existence of these bacteria [7]. In our patients, no microorganisms other than SAG, coming from digestive mucosas, were isolated in aerobic and anaerobic cultures. It is not known whether *Mycobacterium tuberculosis *can enhance the pathogenicity of SAG when they are isolated in the same site of infection, although it has been reported that viridans streptococci may inhibit *Mycobacterium tuberculosis *growth in vitro [8].

Infections due to SAG usually produce an acute or subacute clinical picture [6], but they may also simulate a chronic disease like tuberculosis [9]. From a clinical point of view, it was difficult to discern the contribution of each microorganism to the clinical course of our patients.

Our first case was diagnosed with Addison's disease 2 years previously, when the adrenal tuberculosis went unnoticed. The infection extended from the right adrenal gland to the liver and duodenum, probably causing erosions in the mucosa of the duodenum that favoured the spread of the SAG to the liver and adrenal gland.

Tuberculous psoas and paravertebral muscle abscesses are usually secondary to extension from a vertebral infection, as in our second case. Less frequently, they can be considered primary, without any evidence for other source of infection, as in our third case. SAG aetiology has been described occasionally in psoas abscesses [10], although it has been recently reported as the second most frequent microbiologic isolation in psoas abscesses in another case series from Spain [11]. In that report, all *Mycobacterium tuberculosis *infections were secondary to an adjacent vertebral infection, whereas SAG abscesses had an obvious primary intestinal focus [11]. In our patients, no other source of infection was detected. It is likely that transitory bacteremia, probably originating in the intestine, might have colonized a previously tuberculous lesion.

## Conclusion

In a geographical area with a high prevalence of tuberculous disease, the possibility of dual infection with SAG and *Mycobacterium tuberculosis *should always be considered. Routine processing of bacteriological samples should therefore include the identification of mycobacteria, even if clinical suspicions point in another direction.

## Abbreviations

SAG: *Streptococcus anginosus *group; CT: computer tomography; MIC: minimum inhibitory concentration.

## Competing interests

The authors declare that they have no competing interests.

## Authors' contributions

RR analyzed and interpreted the patient data and was a major contributor in writing the manuscript. CJ analyzed and interpreted the patient data and was a major contributor in writing the manuscript. MR analyzed the data and was involved in drafting the manuscript and revising it critically. CA analyzed the data and was involved in drafting the manuscript and revising it critically. All authors approved the final manuscript

## Consent

Written informed consent was obtained from the patients for publication of this case series report. A copy of the consents are available for review by the Editor-in-Chief of this journal.
